# Case management at an outpatient unit for severe and enduring eating disorder patients at Stockholm Centre for Eating Disorders– a study protocol

**DOI:** 10.1186/s40337-016-0121-3

**Published:** 2016-10-26

**Authors:** M. Molin, Y. von Hausswolff-Juhlin, C. Norring, L. Hagberg, S. A. Gustafsson

**Affiliations:** 1 Department of Clinical Neuroscience, Karolinska Institutet, Stockholm Centre for Eating Disorders, Research & Development, Wollmar Yxkullsgatan 25, S-118 50 Stockholm, Sweden; 2 School of Health Sciences, University Health Care Research Centre, Örebro University, 701 82 Örebro, Sweden; 3 School of Medical Sciences, University Health Care Research Centre, Örebro University, 701 82 Örebro, Sweden

**Keywords:** Eating disorders, Case management, SEED, AN, Quality of life

## Abstract

**Background:**

Patients with severe and enduring eating disorders (SEED) are seriously ill and have a low quality of life. Case management (CM), originally developed for adult patients with severe mental disabilities, has been shown to enhance social functioning and improve quality of life, while reducing the number and length of hospitalizations. In 2014, a special unit based on CM, for patients with SEED (the Eira unit) was started at Stockholm Centre for Eating Disorders, Sweden.

**Method/Design:**

This study aims to investigate if CM can improve SEED patients’ quality of life, and reduce their eating disorder symptoms as well as their health care consumption. Methods for data collection are a semi-structured diagnostic interview, self-report questionnaires, and a qualitative interview. The diagnostic interview and the self-report assessments will be done at start of treatment and at follow-ups after 1, 2, and 3years. The qualitative interview will be conducted 1 year after start of treatment. The study is approved by the ethical review board in Stockholm in compliance with the Helsinki Declaration.

**Discussion:**

CM is a possible new contribution to the treatment methods for SEED. It does not aim at remission, but rather to accept life as it is, and to enhance quality of life in the presence of the ED. This study will investigate the potential benefits of this novel intervention in a special unit for SEED patients.

**Trial registration:**

Clinicaltrials.gov Id: NCT02897622

## Background

Patients with eating disorders (ED) are a challenge for the health care system and the recovery from ED is often slow. Despite massive effort some of the patients do not recover and go on to develop a severe and enduring eating disorder (SEED). Patients with SEED have had an ED for a long time and have often undergone numerous treatments, either voluntarily or compulsory, without recovering from the ED. Owing to the nature of the ED the SEED patients remain seriously ill with severe distress due to social impairments, physical strain and psychiatric symptoms such as anxiety, depression and compulsiveness [1–3]. It is also common that relatives are affected, not only by constant concern, but also because the SEED leads to financial problems that family members have to deal with [4]. Many patients with SEED need long-term sick leave and are heavy consumers of health care, often with long in-patient somatic and psychiatric admissions [2, 5]. SEED patients often feel great and complicating ambivalence towards treatment [6]. Taken together these factors can contribute to a low motivation for treatment, which probably leads to a vicious circle that further complicates treatment and rehabilitation [5]. In the ED field, there is a growing consensus that treatment of patients with SEED needs to be multi-professional, with a focus on improving the patients’ social situation, minimizing medical complications, and enhancing quality of life and independence, rather than focusing one-sided on symptom reduction [3, 7, 8]. It is important to set mutual, acceptable and realistic goals that can be achieved, to do this in collaboration with the patients, and to allow this process a long time [9, 10]. Quality of life for patients with SEED-AN is seriously affected [11] and is as low as the quality of life in severely depressed and schizophrenic patients [8]. Besides the personal suffering of patients and their families, SEED is also associated with high costs for health care and for society in general.

Case management (CM) is a method developed for adult patients with severe mental disabilities such as schizophrenia, other psychoses, personality disorders or serious addiction problems. CM has been shown to improve quality of life [12], enhance social functioning [10], promote ability to live as independently as possible by reducing the number and length of hospitalizations for the patients [13]. CM efforts are individualized and can vary in time from a few months to several years [10]. This study will investigate the potential benefits of such a CM intervention in a special unit for SEED patients.

### Specific aims

The aim of this study is to evaluate a CM model for patients with SEED. Specific questions that the study aims to explore are:

Does entering the CM program lead to:

- Improvement in patients’ quality of life?
- Reduction of patients’ ED symptoms?
- Reduction of patients’ health care consumption and costs?

## Method

### Intervention

At the Stockholm Centre for Eating Disorders in Sweden a new special unit for SEED (Eira) was established in 2014. The staff at Eira consists of one doctor (part time), one social worker and two psychiatry nurses with long experience of ED-treatment.

Before a patient is accepted at Eira, a networking meeting is held, in which the patient is encouraged to bring important people, like family members, remittent or others. Following this meeting, the decision is made whether the patient should be accepted or not.

During the first 5–6 sessions at Eira, the patient’s psychiatric, somatic and social condition is carefully assessed, as well as her/his medical history and previous treatment experiences. It is important for the case manager (i.e. the social worker or one of the nurses) to know the patient and her/his preferences and needs well, in order to be able to coordinate the different caregivers and authorities involved in the patient’s life. The clinical contact between case manager and patient mainly consists of supportive conversations. However, in the “clinical case management” model used in Eira, the case manager also has a treating role, for example by performing social training, somatic controls and family support. The patient’s needs and preferences guide the frequency, place and form of the meetings. Meetings may take place wherever it is deemed appropriate, some meetings may even be in the form of phone calls or text messages.

Another of the case manager’s tasks is to aid the patient in contacts with different authorities and, if necessary, to help with economic issues. It is also important to regularly monitor the patient’s somatic condition.

If the patient is temporarily in need of more intensive somatic or psychiatric care, the case manager can help to plan such an effort. During the period of more intensive care, the case manager maintains contact with the patient by visits and phone calls, and by participating in meetings with the temporary caregivers.

With the patients’ consent, relatives are invited to participate in the CM intervention. Information and support is offered relatives on an individual basis, this also includes underage children. Twice a year, there is also a lecture about the SEED condition for the relatives (without participation of the patients) at Eira. During the lecture participants can ask questions and discuss problems they encounter as SEED relatives.

There is no time limit for the CM intervention. The patient is offered support as long as he/she wants it, independent of degree of ED symptoms or medical condition.

### Participants

The Eira unit can manage 30 patients simultaneously, and all patients included in the CM intervention will be asked to participate in the study. Eira accepts patients who have been suffering from ED for at least 10 years and participated in at least three failed treatment efforts in specialized ED units. Since they have been ill for a long time and since previous treatment efforts have been unsuccessful, patients participating in the study will act as their own controls.

The study is approved by the ethical review board in Stockholm in compliance with the Helsinki Declaration.

### Measures

Methods for data collection are a semi-structured diagnostic interview, a qualitative interview, self-report questionnaires and data from medical records. The diagnostic interview and the self-report assessments will be done at start of treatment and at follow-ups after 1, 2, and 3 years. The qualitative interview will be conducted one year after start of treatment. Data from medical records will be collected retrospectively.

*The Structured Eating Disorder Interview (SEDI)* is a semi-structured diagnostic interview for ED diagnoses according to the DSM-IV. The interview consists of a maximum of 30 and normally about 20–25 questions [14].

*The RAND-36* (also known as the SF-36) measures health-related quality of life (HRQoL) [13]. Changes in the HRQoL over time can be seen by comparing repeated assessments [15, 16].

*The Eating Disorder Examination Questionnaire (EDE-Q)* measures central symptomatic aspects of ED by way of patient’s self-ratings [17, 18].

*The Treatment Satisfaction Scale 2* (*TSS-2*) is a patient-rated assessment of treatment satisfaction in a simple 6-item scale [19]. TSS-2 will be used at all follow-ups.

*The qualitative interview* is semi-structured and consists of three broad themes:

1. The patient’s thoughts about her/his life situation in the year he/she has been at Eira.
2. The patient’s thoughts and reflections about her/his quality of life, and whether it has been affected in the year he/she has been at Eira.
3. The patient’s thoughts about her/his future.

The informants will be asked to talk openly around these themes. The interviewer, who is the same person for all interviews, is a psychologist who is not part of the Eira staff (Table 1).

**Table 1:** Measures over time

Measure	Baseline	1 year	2 years	3 years
SEDI	X	X	X	X
RAND-36	X	X	X	X
EDE-Q	X	X	X	X
TSS-2		X	X	X
Interview		X		

### Outcome

Primary outcome is quality of life. Secondary outcomes are health care consumption and costs, and ED symptoms at 1, 2 and 3 years follow-up.

### Qualitative analysis

The interview will be tape-recorded and transcribed verbatim. After the interview the patients will have an opportunity to review a transcript and to evaluate and comment upon what has been said. The interviews will be analysed with qualitative content analysis according to Hsieh & Shannon [20].

### Evaluation of cost-effectiveness

The cost-effectiveness analysis consists of costs of the CM intervention, changes in quality of life, as well as societal costs such as health care usage and loss of production. The perspective of the analysis will be societal and the time horizon 3 years. The analysis method is going to be cost-utility analysis with health effect expressed in quality adjusted life years (QALY) [21]. The analysis will be complemented with the probability of acceptable cost-effectiveness with different willingness to pay for a QALY [22, 23].

All costs of the CM intervention occur at Eira. The cost for each patient in the study can be calculated by Eira’s total cost divided with each patient’s share of resource utilization based on enrolled time.

QALYs will be estimated based on RAND-36 transformed to SF-6D based on a British preference score [24, 25]. From the measures at baseline, and after 1, 2 and 3 years, changes in QALYs can be estimated. From medical records, changes in cost of health care usage can be calculated. The participants’ employment and change acquisition work rate is followed during the same period based on interviews with participants.

The treatment may also have impact on relatives’ quality of life, costs and earnings. These aspects will not be considered in the analysis.

## Discussion

The treatment of patients with SEED often causes frustration and confusion among ED clinicians, since these patients often have tried “everything” without a lasting positive effect. In our clinical experience this often results in a situation where patients are considered “unmotivated” and dismissed from treatment, which in turn often leads to a symptom deterioration and that patients eventually return to the clinic in need for more acute interventions. As an alternative, clinicians may continue to offer patients interventions that neither therapist nor patient believes is helpful – at least not in a longer perspective. It might be time to re-evaluate what a positive outcome is for these patients.

CM provides an alternative to traditional treatment aiming at symptom reduction. CM aims to improve the patients’ quality of life, enhance their social functioning, and promote their ability to live as independently as possible despite their illness. CM offers unconditional long-term support, without requiring treatment progress. Instead CM stresses stabilization and harm-minimizing interventions, which are assumed to both increase quality of life and social functioning and to reduce the number and length of hospitalizations.

There has been no similar service for patients with ED in Sweden or, to our knowledge, anywhere else. For a long time, CM has been used for patients with serious mental disabilities, such as schizophrenia, other psychoses or personality disorders, as well as for serious substance abuse problems. Since previous studies have shown similarities between SEED patients and patients with schizophrenia [2], it is reasonable to think that CM can be helpful also for SEED. However, we stress that CM is not an intervention for patients with a short duration of illness. For these patients, full recovery should be the main focus. It is also important to point out that CM is not to be seen as a “last resort” or as palliative care. The patients’ goals, life situation and abilities must always be the guiding principle in any intervention, and an important task for the case manager is to always keep an open dialogue with the patients. Although the patients previously have not had a lasting effect of any treatment interventions, this is never to be seen as a “failure”. At another time, in another life situation, the conditions may be better. One of the case managers’ tasks is to guide and help the patients to get access to the right intervention at the right time, and to create the best possible conditions for this intervention.

The primary outcome of this study is quality of life, which will be measured with both quantitative and qualitative measures. It is important to learn more about how SEED patients define quality of life. Is it to have fewer ED symptoms? Better economy? Or is it to be at peace with the ED, without the risk of becoming somatically too ill? Secondary outcome measures are ED diagnosis, ED symptoms, treatment satisfaction and health care consumption. In terms of ED diagnosis, there is no goal of remission with the support of CM. However, it is important to monitor any changes in ED diagnosis and symptoms over time.

The patients’ acceptance of CM at Eira will also be followed up with both quantitative and qualitative measures, to gain an understanding of the patients’ thoughts about the intervention. Perhaps CM will impact the SEED patients’ positively, but there is also a risk that patients will feel more vulnerable and alone when active treatment to reduce symptoms or pushing for weight gain is terminated.

The entire study is uncontrolled and compares conditions before and during the intervention based on an assumption that no changes would occur without the intervention. This assumption creates uncertainty and the sensitivity analysis is therefore particularly important. Especially sensitive to external influences are healthcare usage and productions costs will be considered. They can be affected by changes in the health care organization, enrolment principles and changes in the labour market. Therefore, these conditions will be specifically studied, and in the sensitivity analysis cost-effectiveness will also be estimated without those possible benefits.

In summary, it is our hope that CM, despite its seemingly limited ambitions, will prove to improve living conditions for SEED patients as well as to reduce society’s cost.

## Abbreviations

BMI: Body Mass Index is defined as Weight in kilos/(Length in meters) ^2^
CM: Case management
ED: Eating disorder
SEED: Severe and enduring eating disorder
